# SUMMER Trial: mesh versus suture repair in small umbilical hernias in adults—a study protocol for a prospective randomized double-blind multicenter clinical trial

**DOI:** 10.1186/s13063-021-05366-7

**Published:** 2021-06-22

**Authors:** M. Melkemichel, S. Bringman, G. Granåsen, B. Widhe

**Affiliations:** 1 Department of Surgery, Södertälje Hospital, Södertälje, Sweden; 2 Department of Clinical Sciences, Danderyds Hospital, Karolinska Institutet, Stockholm, Sweden; 3 Department of Clinical Science and Education, Södersjukhuset, Karolinska Institutet, Stockholm, Sweden; 4 Department of Public Health and Clinical Medicine, Umeå University, Umeå, Sweden

**Keywords:** Small umbilical hernia, Mesh repair, Suture repair, Recurrence, Randomized clinical trial

## Abstract

**Background:**

Small umbilical hernia repair is one of the most common surgical performances in general surgery. Yet, a gold standard procedure for the repair is still lacking today. There is an increasing evidence that mesh could be advantageous compared to suture repair in lowering recurrence rates. An additional important question remains with regard to the optimal anatomical positioning of the mesh. We hypothesize that the use of an onlay mesh in small umbilical hernia defects can reduce recurrence rates without increasing the complications compared to a simple suture repair.

**Methods:**

A prospective, national, multicenter, randomized, double-blind clinical trial comparing a standardized 4 × 4 cm onlay mesh to a conventional suture repair will be conducted. A total of 288 patients with a primary elective umbilical hernia ≤ 2 cm from 7 participating Swedish surgical centers will be enrolled. Intraoperative randomization will take place using a centralized web-based system resulting in total allocation concealment. Stratification will be done by surgical site and by defect size. Trial participants and follow-up clinical surgeons will be blinded to the assigned allocation. The primary outcome assessed will be postoperative recurrence at 1 and 3 years. Secondary outcomes assessed will be postoperative complications at 30 days and pain 1 year after surgery.

**Discussion:**

Currently, there has been no randomized clinical trial comparing the recurrence rates between an onlay mesh repair and a simple suture repair for small umbilical hernia defects. How to best repair a small umbilical hernia continues to be debated. This trial design should allow for a good assessment of the differences in recurrence rate due to the large sample size and the adequate follow-up. Surgeons’ concerns surrounding optimal anatomical positioning and fear for larger required dissections are understandable. A small onlay mesh may become an easy and safe method of choice to reduce recurrence rates. Guidelines for small umbilical hernia repairs have stressed the need for reliable data to improve treatment recommendations. We can expect that this trial will have a direct implication on small umbilical hernia repair standards.

**Trial registration:**

ClinicalTrials.gov NCT04231071. Registered on 31 January 2020. SUMMER Trial underwent external peer review as part of the funding process.

## Administrative information

**Table:** 

Title {1}	SUMMER Trial: Mesh versus Suture repair in small umbilical hernias in adults: A study protocol for a prospective randomized double-blind multicenter clinical trial
Trial registration {2a and 2b}.	SUMMER Trial. ClinicalTrials.gov Identifier: NCT04231071. Registered on 31 January 2020
Protocol version {3}	Version 2.0 Dated 24 February 2021
Funding {4}	The trial has research funding from Region Stockholm, ALF and Region Stockholm with Karolinska Institutet in the form of a Medical Resident Research funding grant. The funding sources had no involvement in designing or conducting the trial. There is no commercial sponsoring.
Author details {5a}	^1^ Department of Surgery, Södertälje Hospital, Södertälje, Sweden. ^2^ Department of Clinical Sciences, Danderyds Hospital, Karolinska Institutet, Stockholm, Sweden. ^3^ Department of Clinical Science and Education, Södersjukhuset, Karolinska Institutet, Stockholm, Sweden. ^4^ Department of Public Health and Clinical Medicine, Umeå University, Umeå, Sweden
Name and contact information for the trial sponsor {5b}	Region Stockholm, Department of Surgery, Södertälje Hospital, SWEDEN
Role of sponsor {5c}	The sponsor played no part in developing the study design or writing this protocol for submission.

## Introduction

### Background and rationale {6a}

A small umbilical hernia repair in adults is one of the most common general surgical performances in Sweden [1]. Yet, there is still no gold standard procedure. Traditionally, these small hernia defects have been repaired with an open suture repair, using either a Mayo’s suture or a simple suture technique [2]. Mesh repairs have been primarily reserved more for larger umbilical hernia defects. However, similar results demonstrating lower recurrence when using mesh to repair groin and incisional hernias have also been observed for small umbilical hernia repairs [3].

All data from previous studies has demonstrated lower recurrence rates using mesh reinforcement in open repairs of small umbilical hernias [4–11]. These limited retrospective hand-full published studies reported recurrence rates for suture repair of 4–15% compared to much lower rates for mesh repair of 0–5%. However, only two of them are randomized clinical trials, whereas one of them consisted of only 50 patients with a follow-up of 22 months [6]. The other trial was dated 2001 of included hernias both over and under 3 cm with different mesh positioning [11]. Similar results have been published in a Danish nationwide register-based study, which collected data pertaining to 4786 umbilical and epigastric hernia repairs ≤ 2 cm from the Danish Ventral Hernia Database [12]. The reoperation rate for recurrence in the cohort was 2.2% for mesh repair and 5.6% for suture repair. The same authors also investigated the total clinical true recurrence rates, which were surprisingly high at 21% for suture repair and 10% for mesh repair [13]. This confirms that reoperation for recurrence really underestimates the true recurrence rates and the need for further clinical trials.

Recently, in 2018, a large, randomized, double-blind, controlled trial with 300 participants was published, comparing suture to mesh repair in umbilical hernias of 1–4 cm [14]. The trial demonstrated that mesh reinforcement had a significant reducing effect on recurrence compared to only a suture repair. The pre-peritoneal flat mesh positioning used in this trial with a sublay placement could be difficult to implement in an easy way without enlarging the defect in the case of small umbilical hernias. The peritoneum in the umbilical region is often thin and pre-ruptured. The ligaments below can be difficult to blindly dissect loosely for the creation of a space for an inserted flat mesh. Consequently, an onlay mesh placed above the sutured defect can be considered to achieve the same strength to retrain a hernia from recurring as with a sublay placement, but at the same time be safer and easier to perform in small defects. Also, the role of mesh in very small umbilical hernias < 1 cm remains uncertain. The latter is discussed in a recent meta-analysis [15], together with the important debated question regarding optimal placement of the mesh in these small umbilical hernia defects: sublay or onlay? Additionally, Köckerling et al. newly concluded in a registry-based study of small umbilical hernia repairs < 2 cm which included over 30,000 repairs that suture repair was associated with an increased risk of recurrence [16].

Despite the abovementioned advantages with mesh reinforcement, surgeons have certainly still remained reluctant to use mesh in small ventral hernias. A possible explanation could be due to open questions surrounding optimal anatomical positioning and concerns about larger required dissections and the associated increase in the risk of complications. A meta-analysis found an increased risk for seroma in the mesh group (7.7%) compared to the suture repair group (3.8%) [17]. In contrast, another meta-analysis showed a clear benefit of mesh repair in reducing recurrence rates without any difference in the complication rates between mesh and suture repair [18]. The presence of seroma could seem to be slightly too high in the meta-analysis, and the explanation could be that the analyzed studies are heterogeneous to hernia size and to other factors as mesh positioning. For example, the risk of developing a seroma is theoretically higher in larger hernias repaired with a retro-muscular technique, rather than in very small defects, repaired with a small onlay mesh. A small onlay mesh repair still needs some minor underlying subcutaneous dissection for the inserted mesh above the aponeurosis, and as a result, this can increase the risk of seromas compared to a simple suture repair.

As in groin hernia repair, chronic pain has also become an important issue to assess in umbilical hernia repair. It has mainly been investigated in retrospective studies, demonstrating an incidence of chronic pain of 4–20% without any differences attributable to different surgical techniques [19, 20]. In the Danish cohort study, the chronic pain rate was low and similar in small umbilical and epigastric hernias, regardless of whether a mesh or a suture repair was performed [13].

Although many studies argue that mesh reinforcement offers an advantage also in small umbilical hernias to lower the risk of recurrence, the suitable anatomical mesh position for repairing small umbilical hernias is still uncertain. There are currently no randomized clinical trials comparing a simple suture repair to a simple suture repair coupled with a small onlay mesh for small umbilical hernia defects. Guidelines for small umbilical hernia repairs have stressed the need for reliable data to improve treatment recommendations [21]. We can expect that this trial will provide valuable knowledge and have a direct effect on small umbilical hernia repair standards. If results prove superiority in lowering recurrence rates by using an onlay mesh repair without any significant increase in the occurrence of surgical site complications between the study groups, mesh will have to be considered in treatment standards for small umbilical hernia defects. A small onlay mesh repair could become a safe and easy method of choice.

### Objectives {7}

The aim of this trial is to compare recurrence rates 1 year and 3 years after surgery between a simple suture repair and a simple suture repair with a small onlay mesh in elective primary umbilical hernias ≤ 2 cm. We hypothesize that by using a small onlay mesh in the repair of these small umbilical hernia defects, the occurrence of recurrence rates will significantly be reduced (superiority) without it causing higher surgical site complication rates at 30 days or pain rates at 1 year (non-inferiority) compared to a simple suture repair.

### Trial design {8}

**Fig. 1:**
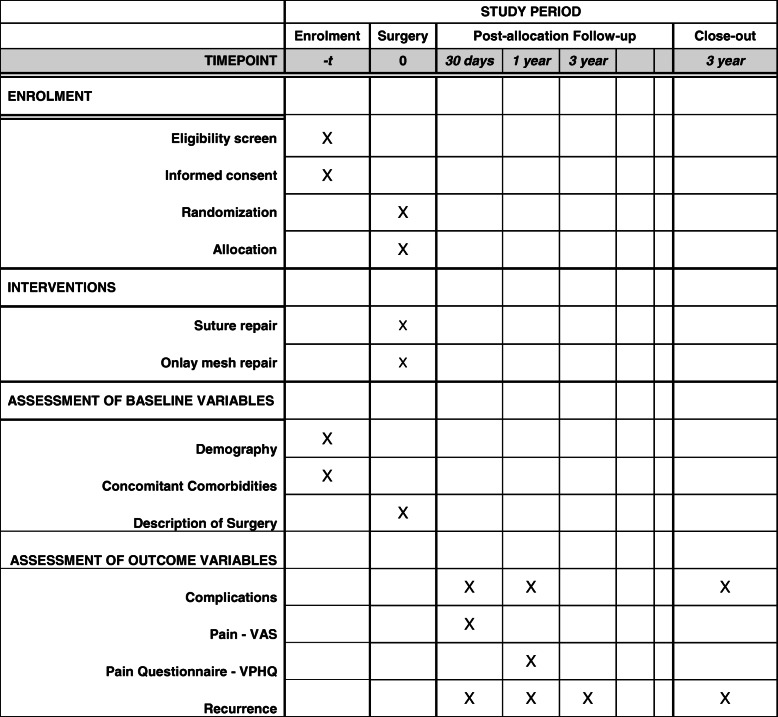
Standards Protocol Items: Recommendations for Interventional Trials (SPIRIT). The SUMMER Trial

The SUMMER (Suture Umbilical Mesh Repair) Trial is the first Swedish national, prospective, parallel-group, superiority, randomized, double-blind, controlled, multicenter trial with patients undergoing open repair for small elective primary umbilical hernias ≤ 2 cm. A web-based online central randomization method with a 1:1 ratio for the two study groups will be applied. The randomization will be performed intraoperatively following the measurement of the defect; the small umbilical hernia defect will be closed with either a simple primary suture repair or with an attached small, flat only mesh on the sutured defect. Trial participants and follow-up clinical surgeons will be blinded to the assigned allocation. Participants will be visiting the outpatient clinic at 30 days, 1 year, and 3 years after surgery for the assessment of the outcomes. Standard Protocol Items: Recommendations for Interventional Trials (SPIRIT) is presented in Fig. 1.

## Methods: participants, interventions, and outcomes

### Study setting {9}

Participating units are currently 7 surgical departments in Sweden with a special interest in hernia repair, with Södertälje Hospital leading the trial. Co-sites are the surgical department at Danderyds Hospital, Norrtälje Hospital, Enköping Hospital, Sophiahemmet/GHP, Mora Hospital, and Frölunda Hospital. Depending on the rate of inclusion, additional centers may be included in the trial.

### Eligibility criteria {10}

A total of 288 adults (> 18 years) will be included in the trial. Patients are recruited to participate in the study by the surgeon during the clinical outpatient meeting if there is an indication requiring surgery. If patients meet all the inclusion criteria and none of the exclusion criteria, they will be invited to take part in the study. All potential trial participants are provided with oral and written information and need to provide written informed consent during the inclusion in the trial prior to randomization and surgery. An umbilical hernia in this trial is defined according to the European Hernia Society definition as a primary midline abdominal wall defect from 3 cm above to 3 cm below the umbilical [22]. Paraumbilical and umbilical hernias are used interchangeably in the SUMMER Trial. This definition has been commonly used in previous studies, which allows a comparison of the results with earlier and future publications on this type of hernia. The inclusion and exclusion criteria are presented in Table 1.

**Table 1:** Inclusion and exclusion criteria

Inclusion criteria	Exclusion criteria
• Elective surgery of a primary umbilical hernia with a defect ≤ 2 cm that has been measured clinically or with radiology	• Umbilical hernia with a defect > 2 cm measured clinically, with radiology or with a ruler intraoperatively
• Age > 18 years	• Multiple defects
• Patients with oral and written informed consent	• Incisional hernia: previous surgery in the area of the operation
	• Recurrent umbilical hernia
	• Epigastric hernia
	• Another operative procedure at the same time
	• Pregnancy
	• Infected wounds
	• Acute operation (incarcerated hernia)
	• BMI > 35 kg/m^2^
	• Ascites
	• Immunosuppression
	• Anticoagulant treatment
	• Connective tissue disorder

### Who will take informed consent? {26a}

The surgeon at the outpatient clinical setting will obtain informed consent from the potential trial participants after the surgeon has ensured that the potential participants have read and understood the written information about the trial. The written information will be sent to the potential participants in advance. The surgeon will check that the potential participants have understood the parts concerning the benefits and risks of participation and will also ensure that the potential participants accept that the treatment will be allocated at random with blinding at the follow-up time.

### Additional consent provisions for collection and use of participant data and biological specimens {26b}

No additional consent provisions are asked from the potential participants except for being part of the SUMMER Trial protocol.

### Interventions

#### Explanation for the choice of comparators {6b}

There is an increasing evidence that mesh reinforcement, compared to a simple suture repair, could be advantageous to lower the high recurrence rates also in smaller umbilical hernias. However, we are still not certain if small umbilical hernias can truly benefit from a mesh repair compared to a suture repair. This is the explanation for choosing the method of one mesh repair compared to a suture repair in the SUMMER Trial. Also, an important question is in which anatomical plane the mesh should be placed. The investigators hypothesize that the use of *an onlay mesh* in small umbilical hernia defects can reduce recurrence rates without increasing postoperative complications compared to a simple suture repair. An onlay mesh placement above the sutured defect can be considered to achieve the same strength to retrain a hernia from recurring as a sublay placement. But at the same time, it is easier to perform in small defects. Surgeons have practiced the onlay-mesh repair for several years, and the comparator with the simple suture repair with a continuous suture is well established in Sweden. Both methods are easy to adopt, and the learning curve of the techniques is considered to be minor without introducing bias to the trial.

#### Intervention description {11a}

At the study initiation, all surgeons participating in the study that will include and operate on patients will be given an oral presentation on the trial and the online electronic data capture software REDCap. The hernia repairs in the SUMMER Trial will be performed by consultants and residents in general surgery. The level of surgical degree will be registered. Only residents that have previous approval from their supervising consultants to perform small umbilical hernia repairs independently are allowed to perform repairs in the SUMMER Trial. All the surgeons will receive a demonstration of the surgical technique by the principal investigator to ensure that they all use the same standardized techniques for the suture repair and the mesh repair described in the protocol. The operation is performed under general anesthesia. No antibiotics are given in any group. Mayo’s hernioplasty of the defect will not be allowed in this trial.

**Fig. 2:**
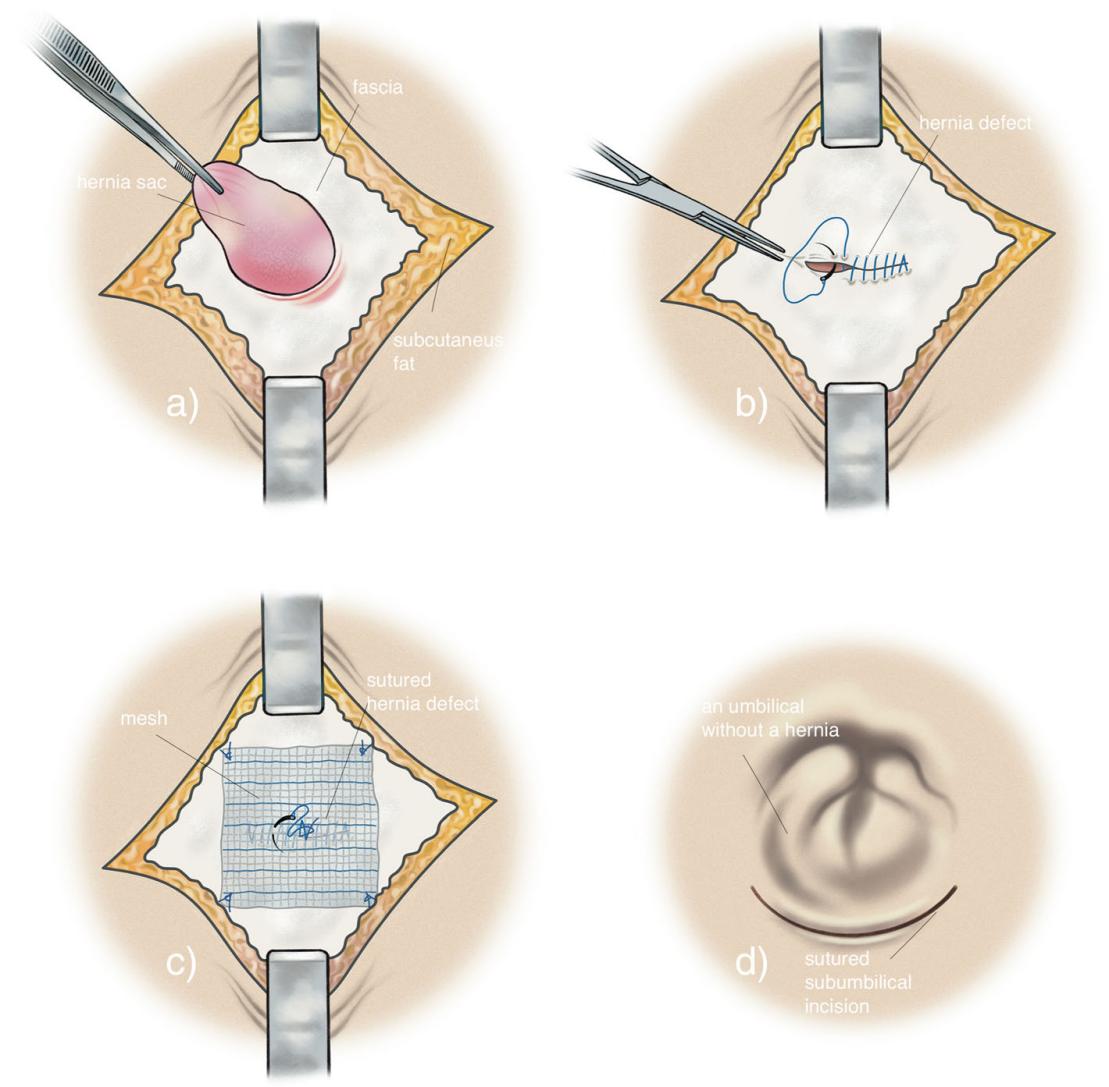
**a** Dissection of the hernia sac. **b** A continuous suture repair. **c** A continuous suture repair with a 4 × 4 cm Onlay Ultrapro Advanced™ mesh on the closed defect, illustrating the 5 single sutures. **d** Closure of the subumbilical surgical incision

*In the controlled sutured group*, the surgeon will perform an open incision in the umbilical area followed by dissection of the hernia sac (Fig. 2a). The largest hernia defect diameter will be measured with a ruler, and patients will then be randomized intraoperatively to either suture or mesh repair. The surgeon will then perform a suture repair with a continuous non-absorbable monofilament suture 2/0 of the aponeurosis defect. The defect will be sutured in the transversal direction, beginning with a start-knot and ending with a stop-knot (Fig. 2b).

*In the intervention onlay mesh group*, the operation will be performed initially as above. The subcutaneous tissue will then be dissected from the aponeurosis so that the surgeon can apply a 4 × 4 cm Ultrapro Advanced™ (© 2018 Ethicon Inc., part of the Johnson & Johnson family of companies, Germany) mesh to the site of the defect that has been closed. The mesh will be fixated with a single non-absorbable monofilament suture 2/0: first, one in the center of the mesh and then one in each corner in a transversal direction to prevent the risk of nerve entrapment. In total, 5 single sutures will attach to the mesh (Fig. 2c). Ultrapro Advanced™ is a lightweight composite polypropylene mesh with an absorbable monofilament poliglecaprone 25 component. The weight is 71 g/m^2^ at implantation and ~ 39 g/m^2^ after absorption. The shrinking of the mesh is ~ 5%.

If the surgeon creates an opening in the umbilical skin during the procedure, the patient will be excluded from the study. In both study groups, an absorbable monofilament suture 3/0 or 4/0 will be used to affix the umbilical skin to the aponeurosis. If the hernia involves the stalk, it needs to be detached. The stalk or the residual of it is re-attached with an absorbable monofilament suture 3/0 or 4/0 to the aponeurosis. If an onlay mesh has been inserted, the umbilical stalk will have contact with the mesh. Closure of the skin will be done with the same type of suture with an intra-cutaneous running suture (Fig. 2d). Finally, the same type and size of the bandage will be placed on the wound.

#### Criteria for discontinuing or modifying allocated interventions {11b}

There are no criteria for discontinuing or modifying allocated interventions. Participants may choose to stop being part of the trial whenever they want to without any reason. Withdrawal of informed consent before the operation will not affect the patient’s entitlement to receive treatment. The surgeon and the patient will then together consider what treatment will be provided for the small umbilical hernia. Usually, suture repair is still the standard care.

#### Strategies to improve adherence to interventions {11c}

None, as the trial participants will have already been operated on and given an intervention that cannot be changed.

#### Relevant concomitant care permitted or prohibited during the trial {11d}

No special provisions.

#### Provisions for post-trial care {30}

None, beyond standard care of patients in Sweden. Participants will be treated the same as any other patients in the healthcare system. The trial participants will have benefit from the Swedish National Patient Insurance System that compensates for any harm that may arise in the trial.

### Outcomes {12}

The primary outcome assessed will be whether a small onlay mesh in the repair of primary umbilical hernias ≤ 2 cm reduces the risk of recurrence compared to a simple suture repair 1 year and 3 years after surgery. Patients will be investigated for recurrence at an outpatient clinical exam via a physical examination of the abdomen by the investigating surgeon following a standard medical assessment. If there is any uncertainty of a recurrence, a computed tomography (CT) scan of the abdomen with a Valsalva maneuver will be performed.

The secondary outcomes assessed will be to compare the two groups of patients with regard to surgical postoperative complications and pain. The postoperative complication rate at 30 days after surgery will be investigated for the presence of a seroma, hematoma, or wound infection. A seroma is defined as an accumulation of clear fluid in the surgical field. A hematoma is defined as an accumulation of blood in the wound area. An infection is defined as a surgical site infection (SSI). The postoperative complication will be graded according to the Clavien-Dindo classification [23]. Grade ≥ 1 is defined as a presence of a postoperative complication. These findings will be investigated clinically with a physical examination by the investigating surgeon following an ordinary medical assessment. If there is any uncertainty of a postoperative complication, a CT scan of the abdomen will be performed. The intensity of postoperative local pain will be assessed with the VAS scale at 30 days. The postoperative pain rate 1 year after surgery will be assessed by the Ventral Hernia Pain Questionnaire (VHPQ). This questionnaire is considered to be a reliable and validated tool to assess pain after ventral hernia surgery [24].

#### Participant timeline {13}

The participant timeline is presented in Fig. 1.

#### Sample size {14}

Taking the previous report’s recurrence rates into consideration, we have predicted and assumed a 12% recurrence rate in the suture group and 3% in the mesh group. In order to detect a difference in the recurrence rate of 9 percentage points after 3 years, a sample size of 288 (144/group) will be required to achieve a power of 80% at a significance level of α = 0.05, allowing for a dropout frequency of 10%.

#### Recruitment {15}

Umbilical hernias are very common in outpatient clinical settings, and therefore, we have confidence in achieving the required number of participants. Despite the COVID-19 pandemic, we have already managed to include 150 trial participants within a 12-month period of unregular healthcare. At each surgical center, a devoted site investigator will identify potential trial participants from the submitted referrals to the site before the outpatient clinical visit. Written information about the trial will be sent out in advance to the potential participants. The site investigator will also continue to motivate the site to recruit the expected number of patients.

### Assignment of interventions: allocation

#### Sequence generation {16a}

The randomization will take place intraoperatively in each hospital, after the inclusion of the patient, by the operating surgeon, directly following the measurement of the hernia defect. If the hernia defect is ≤ 2 cm, the patient has met all of the inclusion criteria and none of the exclusion criteria, and the patient will be randomized during surgery to one of the two operation techniques. Patients will be allocated within the software, using computer-based pre-generated randomization lists. The randomization will be conducted using block randomization with a 1:1 relationship between the two procedures, stratified by surgical site and defect size (≤ 10 mm and > 10 mm).

#### Concealment mechanism {16b}

Randomization will utilize a web-based central randomization system. All random sequences were checked for correctness prior to recruitment. Total concealment of the allocation will be achieved by 4 or 6 block random sequence generation.

#### Implementation {16c}

The biostatistician has generated the allocation sequence. The surgeons will enroll participants in the trial and also assign the participants to interventions by randomizing participants intraoperatively through the online electronic data capture software REDCap.

### Assignment of interventions: blinding

#### Who will be blinded {17a}

Both participants and outcome assessment surgeons will be blinded to the assigned allocation at each of the following follow-ups. The operating surgeon, who is unblinded, will not perform the follow-up visits. The operating surgeon will record in the patient’s hospital health journal that the procedure that has been done according to the SUMMER Trial without specifying if a suture repair or a suture repair with an onlay mesh has been performed. The repair that was performed is then registered in REDCap intraoperatively. This information in REDCap will be locked and hidden for the follow-up clinical investigator. The surgeon will also record the allocated intervention in a separate paper document (not attached to the participant’s hospital health record), which the secretary will keep securely in a separate folder.

#### Procedure for unblinding if needed {17b}

Unblinding is only approved in the case of recurrence or a serious postoperative surgical site infection to allow for an adequate re-operation. At this point, the outcome for this patient has been reached. Unblinding will also be allowed if informed consent is withdrawn. If necessary, there are several procedures for revealing a participant’s allocated intervention in this trial. The allocated intervention can be found in REDCap, but only by the principal investigators. Also, the folded separate paper document folder at each site can be obtained by the secretary.

### Data collection and management

#### Plans for assessment and collection of outcomes {18a}

Demographics and comorbidities of the trial participants will be collected at baseline by the surgeon at the outpatient visit prior to surgery and allocation. Assessment of the outcomes after surgery will be blinded to the allocation, and procedures are described in detail under section 12. Data collection of the outcomes will be completed by a surgeon at the 30-day, 1-year, and 3-year outpatient follow-up visits. At each visit, a palpable physical examination of the trial participant’s abdomen will be performed by the investigating surgeon following a standard medical assessment, and the outcome will be registered in REDCap. The Ventral Hernia Pain Questionnaire will be completed at the 1-year visit to assess pain. The schedule of enrollment, interventions, and assessments is provided in Fig. 1.

#### Plans to promote participant retention and complete follow-up {18b}

None, beyond normal encouragement to visit the outpatient clinic for the follow-ups and motivate the trial participants that the trial results can really benefit umbilical hernia patients in the future.

#### Data management {19}

When participants are included in the trial by the surgeon, after oral and written consent, baseline data will be entered into REDCap by the surgeon. All trial participants will be given a trial number which will be used on all case report forms for that participant within the framework of REDCap during the randomization and the follow-up registration. Only people involved in the trial and authorized by the principal investigators will have access to REDCap and to randomize during surgery via username and passwords specific to each surgical center. Each surgical center will only have data available in REDCap for the participants included at its center and will not have the ability to influence or change any of the data.

#### Confidentiality {27}

Only the above authors will have access to all patient-identifying data, ensuring data protection and preventing unauthorized transportation of data. All person-related data is kept strictly confidential and will be handled in accordance with the European General Data Protection Regulations during and after the trial has ended.

#### Plans for collection, laboratory evaluation, and storage of biological specimens for genetic or molecular analysis in this trial/future use {33}

None

## Statistical methods

### Statistical methods for primary and secondary outcomes {20a}

All analysis will be performed, and data will primarily be presented for the intention-to-treat study population. A corresponding per-protocol analysis will also be performed, and the results from this analysis will be attached as a supplement in the final publication. Statistical tests for the primary endpoint will be two-sided using a significance level of α = 0.05. The primary endpoint will be the recurrence rate observed during a follow-up period of 1 and 3 years and will be analyzed using a mixed logistic regression model with dummy variables specifying each time of follow-up. The primary analysis will be adjusted for fixed effects: defect size and body mass index with a random intercept for each site. Both adjusted and unadjusted results will be presented. Reciprocal Kaplan-Meier curves will be generated to illustrate time to recurrence. Statistical tests for the secondary endpoints will be one-sided using a significance level of α = 0.05. Secondary outcomes that will be analyzed by comparing the treatment groups are postoperative complications 30 days after surgery (Clavien-Dindo scale) and postoperative pain 1 year after surgery (assessed with Ventral Hernia Pain Questionnaire). Postoperative complications and postoperative pain will be analyzed using ordinal logistic regression. The presence of postoperative complications will be analyzed using binary logistic regression. Secondary analyses will be adjusted for fixed effects: defect size and body mass index with a random intercept for each site.

### Interim analyses {21b}

There will be no planned interim analyses.

### Methods for additional analyses (e.g., subgroup analyses) {20b}

At this time, there are no planned additional subgroup analyses. However, if this is reconsidered during the trial, it will be stated in the statistical analysis prior to data lock.

### Methods in analysis to handle protocol non-adherence and any statistical methods to handle missing data {20c}

A comparison of possible discrepancies between the results of the intention-to-treat analysis and per-protocol analysis will be included as part of a sensitivity analysis. In case of a dropout of more than 10% at the 1-year and 3-year follow-up, a sensitivity analysis will be made by comparing the results from the patient’s recorded outcomes at follow-up with a corresponding analysis made by using multiple imputations.

### Plans to give access to the full protocol, participant level-data, and statistical code {31c}

This manuscript is the full protocol. Anyone interested in other participant-level data or statistical code can contact the corresponding author. The uncoded data and statistical code will be uploaded to the ELN system—the secure electronic database of research notebooks, logbooks, and research documentation maintained by Karolinska Institutet.

### Oversight and monitoring

#### Composition of the coordinating center and trial steering committee {5d}

At each participating surgical center, a responsible surgeon performing the site investigator role, will ensure and agree to lead the SUMMER Trial in accordance with the terms of the trial’s clinical study protocol, ethical standards of national research, the latest version of the Helsinki Declaration, and the Good Clinical Practice guidelines. The site investigator at each surgical center will motivate others at the site to recruit the expected number of patients, assist with all trial-related questions, and ensure that the surgeons at the site are collecting and reporting high-quality data while protecting the participant’s personal data. Monthly reports will be given to the principal investigators who will give the trial updates every 3 months.

#### Composition of the data monitoring committee, its role, and reporting structure {21a}

In this trial, a data monitoring committee is considered not to be needed, since the entire trial can be adequately monitored by the online electronic data capture software REDCap. The site investigators at each surgical center will oversee the trial safety and monitor trial progress with regard to recruitment and follow-up. The principal investigators will have a regular contact with each site investigator to ensure that the SUMMER Trial is led in accordance with the trial’s clinical study protocol.

### Adverse event reporting and harms {22}

The SUMMER Trial involves treatments which are well established in the clinical practice for individuals requiring umbilical hernia repair. All early complications in relation to the surgical procedures will be documented and registered within 30 days. Participants will be requested to only contact the surgical unit if a postoperative complication is noted before the 30-day visit. The site investigator at the surgical center will be instructed to report to the principal investigator if an alarming accumulation of postoperative complications is noted. The principal investigator will in that case assess with an uninvolved surgeon whether the trial has to be stopped prematurely.

### Frequency and plans for auditing trial conduct {23}

The principal investigators will have a regular contact with each site investigator to ensure enrollment, recruitment, correct data entry, and randomization by REDCap and that the follow-ups are in accordance with the SUMMER Trial protocol.

### Plans for communicating important protocol amendments to relevant parties (e.g., trial participants, ethical committees) {25}

All protocol modifications prior to inclusion or follow-up will be notified to the ethical committee, and approval of a new protocol version will be required prior to inclusion or follow-up of the trial participants. The protocol amendments will be communicated to relevant parties such as trial participants, site investigators, trial surgical centers, and ClinicalTrial.gov.

### Dissemination plans {31a}

The trial results will be presented at both national and international conferences and published in peer-review journals. The investigators will also discuss the trial results with healthcare professionals within the research area and relevant patient groups that can benefit from the results.

## Discussion

This large, national, multicenter trial is aiming to answer the question whether small umbilical hernia repairs can benefit from using a small onlay mesh to lower the recurrence rates compared to only a simple suture repair. Up until now, the benefits of mesh in these small umbilical hernia repairs have been debated, with evidence that is based on a few small, heterogeneous clinical trials. To our knowledge, this is the only registered trial comparing the onlay mesh method to a simple suture repair. We know from clinical experience that surgeons, despite new data supporting the advantages of mesh, may still be reluctant to use mesh for small hernia defects. A possible explanation could be due to complicated optimal mesh positioning considerations and a perceived increase in the risk of complications. Therefore, if successful, this trial may offer surgeons conclusive evidence that a small onlay mesh repair can be easy, safe, and superior to a simple suture method of repairing small umbilical hernia defects.

The trial population was chosen to be representative of the broader patient population. All patients with an umbilical hernia, despite the initial described size, are evaluated for eligibility to be enrolled in the study at the participating surgical centers. The specific aim is to investigate the treatment for small umbilical hernias ≤ 2 cm specifically, since the mid-sized hernias (2–4 cm) are presumed to already be performed with a mesh reinforcement anyway as part of standard treatment protocols. The exclusion criteria were set up to give an adequate balance between the randomized groups and to only investigate primary elective umbilical hernias. Patients with rare co-morbidities that can affect the outcomes or risk becoming overweight in one study group will be excluded.

The outcomes measured in this trial reflect the concerning issues in using an onlay mesh in umbilical hernia repairs: recurrence, pain, and surgical site postoperative complications. The trial design will allow for good detection of differences in recurrence rate due to the large sample size and sufficient long-term results extending to 3 years. For the secondary outcomes assessment, the 30-day postoperative complications will be investigated in accordance with an international system for grading complications. Furthermore, a validated pain questionnaire for ventral hernia repairs was chosen to assess pain 1 year after surgery. Other questionnaires were considered for use in this trial but were not chosen due to the increased efforts required to conduct the trial at several different surgical centers. The multicenter design reduces distortions related to individual surgeons’ experience and any surgical center bias. The results can therefore be expected to be generalized and applicable for other routine clinical settings. Neither do we expect a learning curve issue to be associated with the onlay mesh method. The technique is not a new one, and the method will have been demonstrated to all participating surgeons. We expect that the method is easy to adapt and perform.

The use of the online electronic data capture software REDCap will ensure correctly inputted data and a total concealment of the assigned allocation prior to randomization. The status of recruitment will easily be followed in real time through the web and motivate the surgical centers to continue to recruit. Another advantage of the online database will be the feasibility of monitoring the whole study.

Moreover, since umbilical hernias are very common in the outpatient clinical setting, we are confident in achieving the required number of participants. However, the time frame of the inclusion period may be at risk of extension due to the COVID-19 pandemic.

Finally, for ethical considerations, the benefit of what this trial can offer umbilical hernia patients outweighs the risks. The risks associated with using a small onlay mesh in this trial are considered to be of a low frequency and, if so, without any severity. The subcutaneous dissection required in the mesh group can increase the risk of postoperative complication of seroma compared to the group of simple suture repair. However, the dissection is considered to be minor, and seromas are not expected to be significantly more frequent than in the simple suture group. Likewise, by using a composite half-absorbable microporous lightweight mesh, the risk of pain after surgery is expected to be equal in both groups. We believe the risk of recurrence can significantly be reduced by using a small onlay mesh compared to only suturing the defect. As such, this trial is expected to have a direct implication, both nationally and internationally, on the small umbilical hernia repair standards.

## Trial status

This trial protocol version 1.0 was published on ClinicalTrial.gov on 31 January 2020 as approved by the Regional Ethics Review Board in Stockholm, Sweden (diary numbers; 2018/22-65 and 2019/05-608). Version 2.0 is referred to as an addition of other surgical centra (Sophiahemmet/GHP), approved by the Regional Ethics Review Board in Stockholm, Sweden, on 24 February 2021. Recruitment started on 3 February 2020 and is estimated to be completed after 24 months. Currently, approximately 150 participants have been included, and 100 are operated and randomized. Due to the COVID-19 pandemic, there is a risk that the inclusion period will need to be extended.

## Data Availability

All trial data will be uncoded and available at the closure, after statistical analysis, in Karolinska Institutet’s electronic notebook database and can be provided on request.

## Abbreviations

BMI: Body mass index
CT: Computed tomography
COVID-19: Coronavirus disease 2019
REDCap: Research Electronic Data Capture
SSI: Surgical site infection
VAS: Visual analog scale
VHPQ: Ventral Hernia Pain Questionnaire
